# Management of dementia risk factors by memory clinic patients and professionals: Pilot study of the BreinZorg (BrainCare) online platform

**DOI:** 10.1177/13872877261440966

**Published:** 2026-04-15

**Authors:** Lotte S. Truin, Irene S. Heger, Kay Deckers, Rani Knops, Tanya Palsma, Rianne A. A. de Heus, Jurgen A. H. R. Claassen, Marjolein E. de Vugt, Sebastian Köhler

**Affiliations:** 1 Alzheimer Centre Limburg, Department of Psychiatry and Neuropsychology, Mental Health and Neuroscience Research Institute (MHeNs), 5211 Maastricht University, Maastricht, The Netherlands; 2 Department of Geriatric Medicine, Radboud Alzheimer Center, Radboud University Medical Center, Nijmegen, The Netherlands; 3 Department of Primary and Community Care, Radboud Alzheimer Center, Radboud University Medical Center, Nijmegen, The Netherlands; 4 Department of Cardiovascular Sciences, University of Leicester, Leicester, UK

**Keywords:** Alzheimer's disease, mild cognitive impairment, prevention, risk factors, subjective cognitive decline

## Abstract

**Background:**

Brain-healthy lifestyle changes could help reduce dementia risk in individuals with subjective cognitive decline (SCD) or mild cognitive impairment (MCI), but patients and clinicians in memory clinics often lack practical tools to support this.

**Objective:**

To explore the acceptability and usability of BreinZorg (English: BrainCare) in a memory clinic setting. BreinZorg is a co-created prototype consisting of a self-management website and hardcopy conversation aid providing dementia risk reduction information.

**Methods:**

The website includes a lifestyle assessment, personalized module recommendations, practical information on 16 modifiable dementia risk and protective factors (e.g., smoking, sleep) and a goal-setting feature. The hardcopy conversation aid briefly outlines these factors to support clinical risk reduction discussions. This study used semi-structured interviews based on the Program Participation Questionnaire and think-aloud sessions, which were analyzed through thematic content analysis. Participants were individuals with self-reported SCD or MCI (n = 19) and memory clinic professionals (n = 9), i.e., nurses/nurse practitioners, physicians/medical specialists and neuropsychologists.

**Results:**

BreinZorg was generally considered useful, trustworthy, easy to use, and applicable in clinical contexts. The platform's accessible design, nonjudgmental tone, and personalization features were appreciated. Some participants with SCD or MCI, particularly those already interested in brain health, desired more in-depth information. A lack of follow-up on personal goals and misaligned module recommendations after the lifestyle assessment were identified as barriers to sustained engagement.

**Conclusions:**

Memory clinic patients and professionals evaluated BreinZorg as practical, useful, and acceptable for supporting dementia risk reduction in clinical care. Offering more detailed information, integrating tailored follow-up support, and enhancing personalization may strengthen long-term user engagement.

## Introduction

The global prevalence of dementia is expected to rise sharply in the coming decades, making it a public health priority.^1^ Two parallel approaches are currently pursued to decrease future dementia cases: the development of disease-modifying pharmacological treatments and the promotion of lifestyle-based risk reduction strategies. The 2024 report of the Lancet Commission on Dementia Prevention, Intervention and Care estimates that about 45% of all dementia cases are potentially attributable to 14 modifiable risk factors, including physical inactivity, smoking, and social isolation.^2^

Despite this, existing evidence indicates that awareness of lifestyle-based dementia risk reduction remains limited, both in the general public^3–6^ and among those with first signs of cognitive decline,^7^ the latter who face an elevated risk of progressing to dementia.^8–10^ Subjective cognitive decline (SCD) refers to self-reported cognitive decline without measurable deficits and could also encompass worry about cognitive decline, while mild cognitive impairment (MCI) involves impairments in one cognitive domain without significant interference in daily life.^8^ In prior research, individuals with SCD and MCI expressed a desire for more information on dementia risk reduction.^11,12^ This underscores their need for education on this topic, which is essential in empowering them to take proactive steps for a brain-healthy lifestyle.

Memory clinic professionals, e.g., nurses/nurse practitioners, physicians/medical specialists and neuropsychologists, are uniquely positioned to lower this knowledge gap in their regular consultations with individuals with SCD or MCI. However, previous studies have identified several challenges in the memory clinic setting that could hinder effective dementia risk reduction discussions. These include limited time per consult, high workloads, and a lack of (online) tools to facilitate these conversations in a practical and accessible manner.^13,14^

Individuals with SCD and MCI have previously been shown to have a positive attitude towards accessing information on dementia risk reduction through an online platform,^15,16^ but they, together with memory clinic professionals, also expressed a need for written information to enhance accessibility and reinforce key messages.^17^ Previous research identified several critical priorities for the successful development of such tools, including the need for personalization, a user-friendly design, and trustworthy, up-to-date content to ensure the tool's effectiveness and engagement.^15^ Within the field of dementia risk reduction, digital interventions have increasingly been explored to support lifestyle change and self-management, with generally positive findings.^18,19^ However, such interventions have primarily been developed and tested in general populations, with very few specifically developed for individuals with SCD or MCI.^20^ Also, these interventions have largely remained confined to research settings and have not progressed into routine, real-world use.^19^ In contrast, digital interventions have been more extensively developed, evaluated and implemented in other health conditions, such as diabetes and cardiovascular disease, where they indeed have shown potential to support behavior change, self-efficacy and sustained user engagement.^21,22^

Based on this, we co-developed BreinZorg (Engl., “BrainCare”) together with SCD or MCI patients and memory clinic professionals. BreinZorg is an evidence-based online self-management platform complemented by hardcopy materials for discussing lifestyle changes for dementia risk reduction in the consultation room (see Supplemental Material 1). In this pilot study, we aimed to explore how individuals with SCD and MCI and memory clinic professionals evaluated the acceptability, usability and perceived added value of the initial BreinZorg prototype, and to identify opportunities for further refinement to support its future implementation in clinical practice.

## Methods

### BreinZorg (BrainCare)

BreinZorg was developed through a collaborative co-creation process, drawing on diverse perspectives, including insights from our previously conducted needs assessment on dementia risk reduction involving individuals with SCD and MCI and memory clinic professionals, expertise in IT development, and design principles. Using an iterative design approach, the platform was refined in reflective cycles, allowing for continuous evaluation and optimization at each development stage. Finally, our first prototype consisted of a website, a hardcopy conversation aid and three posters (Supplemental Material 1). The website and hardcopy conversation aid were designed for use in a healthcare setting together with a healthcare professional, as well as for independent use by individuals with SCD or MCI, either after being introduced by a healthcare professional or through public access. The BreinZorg prototype served as the basis for evaluation in the current study.

The BreinZorg website (www.breinzorg.nl) consists of 16 modules each providing information on a modifiable risk or protective factor for (progression to) dementia. Modules are structured into three categories: lifestyle (alcohol, cognitive activity, physical activity, obesity, smoking, sleep, social contact and diet), body (cholesterol, diabetes, hearing, coronary heart disease, high blood pressure and kidney disease), and mood (depression and anxiety). The website begins with a short, optional lifestyle assessment, which was based on the “LIfestyle for BRAin health” (LIBRA2) score.^23^ Based on their risk profile, users receive personalized, non-binding module recommendations. Users who do not complete the assessment receive full access to all modules, but do not receive such personalized module recommendations tailored to their individual risk profile and therefore lack guidance on which lifestyle domains may be most relevant for them. Each module starts with an introduction to the specific risk factor and its relevance to brain health. Users then complete an interactive quiz to test their own understanding of the risk factor, followed by in-depth information on the factor's association with dementia risk. Each module concludes with a section encouraging users to set specific and measurable personal goals to facilitate behavioral change. In addition to the modules, the platform provides general guidance on goal setting and a frequently asked questions (FAQ) section addressing common concerns about lifestyle and dementia risk. The website also includes a dedicated section for memory clinic professionals offering practical guidance on integrating the platform into their routine care for patients with SCD or MCI.

To complement the website, a hardcopy conversation aid was developed to provide a concise and visually accessible overview of the three categories and their associated factors. It acts as a practical conversation starter for memory clinic professionals, facilitating their conversations about dementia risk reduction in an approachable and structured manner. Additionally, posters were designed to promote BreinZorg in the waiting room.

### Participants

The study included individuals with self-reported SCD or MCI who had previously visited a memory clinic, as well as memory clinic professionals (e.g., nurses, physicians and psychologists). No further eligibility criteria were applied to allow inclusion of a broad and diverse user group. Most participants were recruited via our prior needs assessment, where they expressed interest in this pilot study. This initial recruitment occurred through Dutch memory clinics, the online Dutch Brain Research Registry,^24^ the Dutch Memory Clinic Network and the Alzheimer Centre Limburg online communication channels. They were supplemented by two newly recruited MCI participants from Maastricht University Medical Center+. A purposive sampling strategy was used, aiming for a balanced representation of individuals with SCD and MCI, as well as a variety of memory clinic professionals. Based on a similar pilot study,^25^ we expected to include 15 to 20 participants for in-depth interviews and 6 to 9 participants for think aloud sessions to achieve data saturation. Eligible participants were invited via email or phone call according to their preference and received an information letter detailing the study. They were given the opportunity to ask questions before providing written informed consent. Demographic information was collected at the start of the interview or think-aloud session. Educational level was categorized according to the Dutch standard education classification of Statistics Netherlands: low (primary education/vmbo/mbo1), intermediate (havo/vwo/mbo2-4), and high (hbo/wo).^26^ The study protocol was approved by the Medical Ethics Committee of Maastricht University Medical Center + (#2023-0046), The Netherlands.

### Interviews

Approximately two weeks prior to the interviews, participants were provided access to the BreinZorg website and received the hardcopy conversation aid and posters by mail. They were instructed to explore the website and were encouraged (but not required) to complete the lifestyle test. As the website is self-paced and not all modules are necessarily relevant for every user, participants were not expected to complete all modules within this two-week period. The aim was to evaluate the website based on engagement with relevant modules, rather than to obtain a full evaluation of the entire module set. Participants were also asked to review the conversation aid and posters critically before the interview. Individual, semi-structured, in-depth interviews were conducted either online (via Teams) or in person, based on the participant's preference. Interviews took approximately one hour. The interview topic guide was based on the Program Participation Questionnaire (PPQ, see Supplemental Material 2).^25^ The original PPQ assesses perceived usefulness, ease of use, content quality, and overall acceptance of an online platform for informal caregivers of individuals living with dementia. In this study, the PPQ items were adapted to reflect the specific content and structure of the BreinZorg prototype, including its modules and delivery format, while still targeting the same underlying constructs. Rather than being used as a standalone quantitative questionnaire, the PPQ items served as structured prompts to guide the interviews. Participants were presented with each item and could optionally rate it on a 7-point Likert scale (1 = ‘completely disagree’, 7 = ‘completely agree’); however, providing a rating was not required. The primary purpose of the items was to facilitate discussion and encourage participants to reflect on specific aspects of the BreinZorg prototype. The qualitative responses elicited by these prompts formed the basis of the analysis. Separate guides were developed for participants with SCD or MCI (29 questions, see Supplemental Material 3) and memory clinic professionals (27 questions, see Supplemental Material 4).

### Think-aloud sessions

In-person think-aloud sessions were conducted to evaluate participants’ initial interactions and experiences with the prototype. This method encourages participants to verbalize their thoughts as they engage with a task, providing real-time insights into comprehension, decision-making processes, and potential barriers.^27,28^ By capturing participants’ immediate reflections with minimal researcher interference, the think-aloud method offers an unfiltered view of thought processes.^29^ It was particularly suited to evaluate participants’ real-time use of the prototype and identify usability challenges. Minimal prompts were used when necessary to maintain the flow of the session (e.g., “What do you think of what you are seeing?”).

### Evaluation of use in clinical practice

Finally, a series of additional, short (15 to 30 min) semi-structured interviews were conducted, either in person or online via Teams, with memory clinic professionals as an initial step to evaluate the use of the prototype in clinical practice. Professionals were instructed to freely use the conversation aid in their daily practice, with the option to include the website and posters, to facilitate conversations about dementia risk reduction, brain health, and lifestyle among patients with SCD or MCI at their memory clinic. Follow-up interviews explored their experiences, including perceived effectiveness, barriers, and facilitators to using the prototype in practice. The topic list can be found in Supplemental Material 5. Two brief questionnaires on perceived usefulness and ease of use where each item was rated on a 7-point Likert scale (1 = ‘completely disagree’, 7 = ‘completely agree’) were administered during the interview to further prompt discussion on the perceived ease of use and usefulness of the materials (see Supplemental Material 6). Both questionnaires were adapted from the Technology Acceptance Model^30^ to the BreinZorg context.

### Data analysis

Initial interviews and think-aloud sessions took place between September and December 2023, and interviews for evaluation of use in clinical practice between June and October 2024. The interviews and think-aloud sessions were conducted by author L.S.T., with authors I.S.H. or R.K. present whenever possible to take field notes. Data collection continued until data saturation was achieved, meaning no new themes emerged, and strong repetition was observed across interviews and sessions.^31^ Interviews and think-aloud sessions were conducted in Dutch, audio-recorded and transcribed verbatim. Quotations were translated into English using Google Translate for reporting purposes. Transcripts were analyzed using ATLAS.ti version 24. The qualitative analysis was guided by a pragmatic epistemological stance, emphasizing practical insights from participants’ experiences to support future iterative improvement of BreinZorg. We employed an inductive thematic analysis approach, chosen for its flexibility in identifying patterns and themes that emerge directly from the data without imposing predefined categories.^32^ The analysis began with familiarization: all transcripts were thoroughly read to gain a first understanding of participant responses. Initial coding was conducted independently by two authors (L.S.T. and I.S.H. or R.K.) without the use of predefined categories or frameworks. This allowed key themes to emerge naturally from the participants’ responses. Following independent coding, the authors compared and discussed discrepancies, reaching consensus on the key findings. Through this process, recurring patterns and key findings were synthesized into overarching themes. These themes were further refined and discussed with all authors to determine the final primary findings. While coding remained inductive, special attention was paid during the analysis of both interviews and think-aloud sessions to identifying verbalized barriers, such as difficulties in understanding, navigating, or engaging with the BreinZorg prototype, along with usability insights, including feedback on the prototype's clarity, relevance, and ease of use.

## Results

Nineteen individuals with SCD or MCI and nine memory clinic professionals participated in different activities of this study. Thirteen semi-structured interviews were conducted with individuals with SCD or MCI and five with memory clinic professionals. In addition, six think-aloud sessions were conducted among individuals with SCD or MCI and three among memory clinic professionals. Finally, four additional semi-structured interviews were held with memory clinic professionals to explore the use of the prototype in a clinical practice setting. Participant demographics can be found in Table 1.

**Table 1.** Participant demographics of individuals with SCD or MCI and memory clinic professionals.

Individuals with SCD or MCI (n = 19)			Memory clinic professionals (n = 9)	
MCI, n (%)	9 (47.4)		Women, n (%)	8 (88.9)
Women, n (%)	7 (36.8)		Mean age, years (SD)	43.7 (10.1)
Mean age, years (SD)	67.1 (7.8)		Profession	
Education			Physician/medical specialist, n (%)	2 (22.2)
Low education, n (%)	1 (5.3)		Nurse/nurse practitioner, n (%)	3 (33.3)
Middle education, n (%)	13 (68.4)		Neuropsychologist, n (%)	4 (44.4)
High education, n (%)	5 (26.3)		Years of work experience at memory clinic	
			One to five years	4 (44.4)
			11 to 20 years	4 (44.4)
			More than 20 years	1 (11.1)

Thematic analysis identified four main themes: the need for layered and in-depth information, the impact of tone and message framing in risk communication, factors that encourage sustainable behavior change, and suggestions for implementing the prototype in practice (Table 2). The themes are presented below, accompanied by illustrative quotes.

**Table 2.** Key findings of the four identified themes.

Theme	Key findings	Perspective reported by
*1. Information needs*	Already familiar content served as a useful reminder and encouraged self-reflection, despite some redundancy.	Individuals with SCD or MCI
	Participants valued the layered and straightforward presentation of basic information but sought more depth through external, reliable, and trustworthy resources.	Both participant groups
*2. Risk communication*	The positive, non-judgmental tone in the prototype was appreciated and made the content more approachable.	Both participant groups
	Participants valued personalized, context-sensitive feedback on the lifestyle test, which could be further enhanced to increase user engagement.	Individuals with SCD or MCI
	Mildly confronting content was accepted when framed constructively, because of its value in promoting awareness and self-reflection.	Individuals with SCD or MCI
	Messaging should focus on empowerment: encouraging to take proactive steps while avoiding victim blaming or overemphasizing individual control on dementia risk.	Both participant groups
*3. Encouraging sustainable behavior change*	Concrete tips and the ability to set personal goals encouraged initial behavior changes.	Both participant groups
	There was a need for further guidance on sustainable lifestyle changes, such as reminders or optional coaching.	Both participant groups
	Involving family members or partners was seen as a facilitator for behavior changes.	Both participant groups
	Participants needed clearer communication on short-term benefits of brain-healthy lifestyle changes, rather than solely long-term dementia risk reduction.	Individuals with SCD or MCI
*4. Implementation*	The combination of the website and hardcopy conversation aid was well received and seen as especially helpful for patients with varying digital skills.	Both participant groups
	The conversation aid was useful for to initiating, structuring and enriching dementia risk reduction conversations in clinical practice.	Memory clinic professionals
	The prototype was regarded as widely applicable, including in contexts beyond the memory clinic, such as primary care.	Both participant groups
	The prototype's credibility was strengthened by being offered through a reputable source, such as a university or health organization.	Both participant groups
	There was a need for a dedicated section offering general guidance on how to cope with memory complaints.	Individuals with SCD or MCI

### Information needs

Participants with SCD or MCI generally had prior knowledge about the link between lifestyle, brain health, and dementia risk reduction, and some found the prototype's content redundant. Still, they noted it served as a useful reminder and encouraged reflection on their own habits. While not new to them, they felt the information could be valuable for others with less background knowledge.

“*I am so immodest as to say that I already know quite a lot about it and live very healthy and do not smoke or drink alcohol and have a good body mass index. So [the information] is useful in the sense that I am doing well, but not that I had to learn much from it. […] But it is good to read again.” (Participant with MCI, female, 63 years, middle education)*

Additionally, participants appreciated the platform's flexibility, which allowed them to select information that aligned with their interests or topics on which they had knowledge gaps and room for improvement. This design minimized the impact of redundant content, allowing them to easily bypass familiar material and concentrate on personally novel information.

“*[The platform] is very complete and clear, and you can do ‘cherry picking’, you don’t have to read everything, but you can just choose a module.” (Participant with MCI, female, 63 years, middle education)*

Most participants with SCD or MCI expressed a preference for even more new and in-depth information, such as a comprehensive exploration of important concepts and mechanisms by which specific lifestyle factors influence the brain or dementia risk, as well as novel insights into dementia risk reduction from scientific research.

“*I thought [the information] was nice, but it stopped soon. […] Sometimes you think; oh, I didn't expect that, and I would like to look further into that. Or what should I think about this, or what should I do about it?” (Participant with SCD, male, 75 years, high education)*

Participants with SCD or MCI sought additional depth, not necessarily integrated into the prototype itself, but available through a reference list of external resources. Participants emphasized that the list should feature reliable and trustworthy sources, such as government agencies, health institutes, or academic institutions, providing users with direct links for further exploration. As one participant suggested:

“*Perhaps consider adding a clickable link to a reliable website or something similar.” (Participant with SCD, male, 75 years, high education)*

Memory clinic professionals shared similar perspectives, emphasizing the importance of clear, accessible information on dementia risk reduction to cater to users with different knowledge levels. They appreciated the website's layered layout and agreed with the idea of offering more detailed information through optional links, while maintaining the platform's simple core design.

“*If you hover over [for example] ‘hippocampus’, that you can click on it and get a kind of add-on information, if you want that. […] If I click on that or if I hover over that, then I can go a bit more in depth, while you keep the basics quite simple.” (Medical specialist, female, 33 years)*

### Risk communication

The tone of the prototype was generally described as positive and non-judgmental, which was appreciated. Our participants acknowledged the seriousness of the subject, but the light-hearted tone made the content more accessible and less overwhelming, particularly for individuals who may already feel burdened by their health challenges. As one participant explained:

“*I think ‘Care that you don’t forget’…, yeah, I find that [slogan] funny. I like it when there's a joke in there. In my opinion, you can make jokes about these kinds of things. […] You know, it's already heavy enough when you start having memory problems. So, I don’t like it when everything is made too heavy.” (Participant with MCI, female, 58 years, middle education)*

On the other hand, some other participants felt the positive framing in fact somewhat downplayed the seriousness of dementia risk and lacked urgency. All participants, but especially memory clinic professionals, therefore agreed on the need for a balanced approach: one that is both informative, urgent and motivating while avoiding a condescending or dismissive tone of voice.

“*Not so forceful, you know? Not that finger-pointing…* *Just more like, ‘this is something you could do to…’ But without instilling fear of illness or, uhm, worsening health. It's really about a kind of positive health approach, and I really appreciate that about the tone of the text.” (Participant with MCI, male, 78 years, low education)*

A few participants felt some content lacked sufficient sensitivity or nuance, describing parts of the text as overly instructive. This reduced the intended positive impact and, in some cases, affected their motivation to engage.

“*I sometimes found it a bit preachy. I mean, there are things in there that I already know. And then I think, ahhhh…* *[…] I also did the [module] on overweight, and it [generally] says being overweight is not good. And then I think, well yeah, hello, yes, yes, yes…* *And I do understand that it needs to be mentioned, but it just comes across as so preachy. […] It doesn't help.” (Participant with SCD, female, 63 years, high education)*

Some SCD or MCI participants found the information on lifestyle and dementia risk mildly emotionally confronting, feeling somewhat vulnerable and concerned about their habits. Memory clinic professionals made similar remarks when reflecting on the potential impact on their patients. Most SCD or MCI participants still wanted information on dementia risk reduction and accepted slightly discomforting content, emphasizing its role in raising awareness and fostering self-reflection. One participant stated:

“*It is of course always confronting. The moment you have to fill in your weight and those things. So, I find it quite confronting. […] But I think that's just how it is. Yes…* *that's just how it is. […] But, it's okay for it to be somewhat confronting and uncomfortable, right?” (Participant with MCI, male, 78 years, low education)*

Both participant groups emphasized the general importance of setting realistic expectations and nuanced messaging that avoids oversimplifying behavioral change. Specifically, blaming the victim and suggesting that individuals can fully control their risk through lifestyle adjustments alone should be avoided, as BreinZorg has done by acknowledging limits to individual control. As one participant stated:

“*That the text does not convey the message that someone is personally responsible for their condition. And by ‘responsible,’ I mean two things: that they are to blame for having [memory problems] and that they can solve it on their own.” (Neuropsychologist, female, 44 years).*

Instead, participants felt the messaging should focus on empowerment: encouraging individuals to take proactive steps while acknowledging that several determinants for dementia remain beyond personal control, as reflected in our prototype. Participants’ user experiences further underscored the need for clear, nuanced communication. SCD or MCI participants generally liked the lifestyle test and module recommendations, but some noted that the test's limited answer options did not fully capture personal context. This led to feedback that occasionally felt oversimplified or misaligned with their own perceptions of their health status.

“*Regarding cholesterol, there's still room for improvement. But that's basically concluded based on how I filled it in. That's nonsense. I mean, is there really room for improvement? […] Yes, but this has been the case my entire life. My GP even told me, ‘That's just how your body works.’ […] I'm not doing anything unusual. […] Sure, improvement might be possible, but it would require living a very strict lifestyle, following a rigid schedule. Maybe I don't even want that. I live very simply now, following normal routines.” (Participant with SCD, male, 78 years, middle education)*

The misalignment sometimes resulted in feelings of uncertainty or mild frustration. Rather than motivating them, it made participants less inclined to engage with the recommendations.

Overall, tone of voice appeared to be an important factor for participants, influencing both their motivation to actively engage with the prototype's content and their willingness to implement the information in their daily lives. However, participants’ responses to tone of voice varied, underscoring that there is no one-size-fits-all.

### Encouraging sustainable behavior change

Some participants with SCD or MCI noted that the information provided in the prototype prompted them to make small, one-time adjustments to their lifestyle, such as adjusting dietary choices shortly after engaging with the content. As one participant described:

*But because of what I have read here, I thought that I could watch my cholesterol a bit more with food. And I was eating cheese the other day, and then I thought: wait a minute! I will [eat] that a bit less. So, it does have an impact, it does something. And it has a positive contribution toward memory problems.” (Participant with SCD, male, 57 years, middle education)*

Providing concrete tips in each module to encourage such changes and formulating personal goals was generally evaluated positively by both participant groups. However, nearly all participants expressed a wish for more guidance on how to consistently and sustainably implement these lifestyle recommendations in daily routine. They highlighted the possibility for more active coaching, noting that hands-on tips are often more effective for lasting change than theoretical discussions alone. One participant described:

“*I did try another lifestyle program where you could exercise for free for three months in [city], and I’m still doing it. So yes, that's something that could really work when provided through healthcare — letting people experience something firsthand.” (Participant with SCD, female, 63 years, high education)*

They further explained how this hands-on approach helped lower the threshold for making long-term changes:

“*For me, it was lowering the threshold [to start a new habit]. I was able to join that other two-year program where both exercise and lifestyle coaching were included. […] We tried different sports in groups, and it was actually fun. Now, I still swim twice a week. And that all started because of that program.” (Participant with SCD, female, 63 years, high education)*

Most participants with SCD or MCI also expressed interest in personal coaching, if users could decide for themselves whether and how to engage with it. Memory clinic professionals, however, identified barriers to implementing coaching due to limitations in both time and available funding within the healthcare sector.

The inclusion of some basic follow-up emerged as a recurring theme in all interviews. Participants appreciated the self-management approach of the prototype and suggested that (optional) follow-ups, such as reminder emails or check-ins by a healthcare provider, could further strengthen motivation and long-term engagement. As one memory clinic professional noted:

“*Maybe there should be some kind of follow-up [after setting a goal]. […] Like receiving a personalized email asking, ‘Hey, how did it go? How did you manage?’. Something like that, just to provide a bit of extra encouragement and accountability.” (Nurse, female, 42 years)*

Another key aspect discussed by all participants was involving the user's social environment in the prototype, such as close family members or partners, who might be affected by the lifestyle changes and could facilitate sustainable behavior change. For example, one participant shared how both she and her partner adapted their eating habits after reading about the benefits of consuming fish and legumes:

“*We will eat fish tonight because we have read several times about it.” (Participant with MCI, female, 63 years, middle education)*

Participants with SCD and MCI expressed that clearer communication regarding the personal, short-term benefits of brain-healthy lifestyle changes could further enhance motivation, rather than merely focusing on long-term risk reduction. One participant stated:

“*What is in it for me? I am making an effort, so what is my [short-term] benefit?” (Participant with MCI, female, 63 years, middle education)*

### Implementation

*Evaluation of materials.* All participants with SCD or MCI were positive about receiving information on dementia risk reduction through our website. They appreciated its accessibility and design for use on both desktop and smartphones, offering flexibility in preferred device choice. Participants also valued concise, digestible hardcopy materials like our conversation aid, which directed them to digital sources for more detailed information. They preferred this format over solely physical documentation, which they described as potentially overwhelming.

“*I’m having surgery for carpal tunnel syndrome — though that's not the point — but I received this big stack of information with all these A4 pages, just a whole packet of text. So, I ended up searching online instead. But if I had been given a flyer with less information and a link to a website, that would have appealed to me more than all those A4 pages I got. […] ““&rdquo; (Participant with SCD, female, 63 years, high education)*

Memory clinic professionals were similarly positive. Although they sometimes expressed concerns about the platform's accessibility for patients with lower digital literacy or advanced age, they frequently emphasized:

“*[Digital information] is the future.” (Professional, nurse practitioner, female, 40 years)*

The combination of our digital and hardcopy materials was therefore particularly well-received. One notable advantage of the conversation aid was its usability for less digitally competent patients.

“*I can imagine that [something digital] can be difficult when you are 80 plus. So, it is also good to have the paper version.” (Nurse practitioner, female, 40 years)*

*Use in clinical practice.* Memory clinic professionals primarily used the conversation aid in their daily practice: to initiate conversations about lifestyle and dementia, visually support these discussions, and refer to more in-depth information on our website. It provided professionals with a framework, helping them facilitate patient self-reflection, enhance engagement, and reduce reliance on verbal communication.

“*It really provided me with a kind of framework. […] We know that talking too much doesn’t always resonate, and we know that the brain is fragile in our setting. At the same time, we know that verbal information isn’t the best way to convey things, yet what we do is talk. So, what I noticed here was that I didn’t have to talk as much; on the other side [of the conversation], there was much more thinking happening, both individually and with their partner. […] And then you can guide them more and adjust things if what they say isn’t correct, but they are doing a lot more themselves.” (Professional, neuropsychologist, female, 44 years)*

Memory clinic professionals noted that the prototype addressed an unmet need. Previously, they sometimes felt unprepared when patients with SCD or MCI inquired about reducing their dementia risk. The prototype now offers easy-to-provide, concrete steps patients can take to minimize their risk.

“*It's definitely good to be able to share something that people can do to help themselves… to potentially influence their cognitive complaints. It's not pleasant to have to say, ‘There's nothing you can do. Good luck.’ […] The [materials] are just very concrete and practical, and I think that's what makes them so useful.” (Neuropsychologist, female, 47 years)*

*Implementation opportunities.* Participants named various opportunities for facilitating the implementation of the prototype, both in and beyond memory clinics. Memory clinic professionals highlighted the prototype's versatility, noting that it should certainly be used by a broad spectrum of healthcare providers treating patients with SCD or MCI, including psychologists, physicians, nurses and lifestyle coaches. All participants emphasized that the prototype could be effectively utilized beyond the memory clinic setting, e.g., in general practice settings, geriatrics departments or other relevant departments in hospitals.

“*I would find it interesting, for example, as part of a follow-up study, to push this more toward primary care. More toward general practices and practice nurses. Because they could definitely use this in their practice with people who don’t yet have dementia, and who could then be triggered to reduce their risk of developing it.” (Nurse practitioner, female, 40 years)*

Participants recommended integrating the prototype within a larger patient organization. They believed this alignment would make the materials easier to locate for individuals with SCD or MCI and their healthcare providers. Centralization was also viewed as a means to address the current fragmentation of information and tools, potentially improving accessibility and reach.

“*BreinZorg is yet another new topic, while I actually think it should fall under Alzheimer Nederland or the Hersenstichting. It's yet another offshoot of everything. […] Not too much fragmentation. Then you end up with all these separate topics that are scattered.” (Participant with SCD, male, 64 years, middle education)*

*Additional needs.* Participants with SCD or MCI appreciated the prototype being provided by a reputable source, such as a university or a health organization, and underpinned by the latest evidence-based findings. This credibility was seen as essential in building trust and encouraging users to explore the prototype.

“*That [the information comes from science] definitely matters to me. Of course, I first go by my own feeling — what do I need? And then I start reading to see what's been written. And it definitely matters to me whether something can be scientifically substantiated. Because anyone can have an experience of what works, but it might not work in the long term.” (Participant with MCI, female, 47 years, high education)*

Lastly, participants with SCD or MCI expressed a need for a dedicated section offering general guidance on how to cope with memory complaints, which could enhance the prototype's uptake. This should include practical tips for managing daily challenges and resources for accepting existing memory problems.

“*But also [information on], for example, you know… How do you deal with [memory problems]?” (Participant with MCI, female, 58 years, middle education)*

“*I am trying to find a way to either sharpen my memory or just to accept it. […] But [information on this] would be nice, like how we are discussing it now, yes.” (Participant with SCD, male, 75 years, high education)*

## Discussion

BreinZorg (BrainCare) is an online, evidence-based self-management platform and hardcopy conversation aid, which was designed to support individuals with SCD or MCI in adopting brain-healthy lifestyle changes and to assist memory clinic professionals in discussing dementia risk reduction. This pilot study aimed to evaluate the prototype's feasibility, usability, and potential for further development towards practical implementation. The prototype was well-received by both individuals with SCD or MCI and memory clinic professionals as a trustworthy resource for information on reducing dementia risk. Furthermore, it was valued for its accessible design, balanced tone, and practical application in clinical conversations. Key areas to enhance user engagement include personalized, context-sensitive feedback (e.g., streamlining module recommendations), integrating general information on managing memory complaints, and further guidance on behavior change through reminders (e.g., via e-mail) or optional coaching (potentially including an in-person component).

Many participants positively evaluated the platform's content as a helpful resource and reminder to reflect on current lifestyle habits. Simultaneously, individuals with SCD or MCI desired new and more comprehensive information and access to reliable external references for further exploration of dementia risk factors. Our findings are in line with previous research focusing on Dutch individuals with SCD, which described a similar need for more new, in-depth information on brain health and lifestyle among participants testing an online program on this topic.^20^ While our prototype was designed to support self-management of dementia risk factors, participants with SCD or MCI sought additional, practical tips to manage daily cognitive challenges and accept their condition. This reflects findings from a systematic review of patient perspectives on MCI, which concluded that individuals with MCI benefit from support and guidance on coping with everyday cognitive challenges and emotional adjustment to the diagnosis.^33^ Including such additional content would provide a more holistic approach to supporting individuals with SCD or MCI. Both groups of participants regarded the prototype as originating from a reputable source (a university), which promoted credibility and further supported usage. In line with our findings, the author's authority has been previously linked to increased credibility and uptake of web-based health information.^34^ Additionally, participants saw embedding the prototype within established patient organizations, such as the national Alzheimer's Society, as a way to further enhance its visibility, accessibility, and trustworthiness. This echoes prior research among adults in the UK, indicating that the integration of health apps within trusted organizations can strengthen user engagement and facilitate implementation.^35^

All participants appreciated the platform's flexibility and user-friendly design, allowing them to engage with the content at their own pace and select modules aligned with their interests. The module recommendations from the self-reported lifestyle test and goal-setting options promoted personalization and user autonomy. These were key features that enhanced user engagement, although they could have been further optimized. Indeed, personalization and user autonomy were found to be critical facilitators for using eHealth applications among different patient groups.^20,36–38^ While participants felt encouraged by the platform to take first steps, a need for follow-up or optional coaching, sometimes with a preference for personal or face-to-face contact, was expressed to encourage sustained behavioral changes in users. This is consistent with findings from other health interventions that emphasized the role of ongoing support in achieving long-term, health-related goals and improving adherence.^21,39–42^

Effective risk communication is vital in dementia risk reduction strategies, and the tone of voice can significantly influence motivation and engagement. Prior research has shown that overly directive or threatening communication can lead to guilt and avoidance, but balanced, nonjudgmental communication fosters trust and compliance.^43^ However, oversimplified communication may also undermine effectiveness by downplaying complexity and nuance.^44^ This highlights the need for a careful balance between clarity, accuracy, and motivational tone. In agreement with this, many of our participants appreciated the prototype's nonjudgmental tone while also recognizing the value of the occasionally mildly confronting content, as it fostered awareness and induced self-reflection. Acknowledging that both modifiable and non-modifiable factors influence dementia risk helped prevent blame and supported more realistic expectations, as also emphasized in the broader literature on ethical dementia risk communication.^45^

When designing interventions for dementia risk reduction, they should be accessible to a wide range of individuals, including those with limited digital skills. This group is often underserved by digital-only health interventions, highlighting the importance of designing more inclusive solutions.^46^ Our findings are adding to this evidence. Memory clinic professionals were particularly concerned that digital resources on dementia risk reduction alone might not effectively reach their older patients with limited digital literacy. Both participant groups praised our prototype's dual format, which, according to them, improved accessibility and enhanced its inclusiveness. Additionally, professionals mainly used the conversation aid due to its practicality, highlighting another benefit of our format approach.

### Strengths and limitations

This study has several strengths. First, including individuals with SCD and MCI, along with a diverse array of memory clinic professionals, all from various regions of the Netherlands, offered broad perspectives and experiences regarding the prototype. Second, the qualitative nature of this study (in-depth interviews and think-aloud sessions) allowed a thorough exploration of those perspectives and experiences. Third, the prototype was co-created and iteratively developed with individuals with SCD and MCI and memory clinic professionals, ensuring that it closely aligned with their needs and preferences. Involving additional stakeholders throughout the development process further enhanced its practical relevance and usability, which may facilitate future implementation in real-world clinical settings.

However, some limitations should be considered when interpreting our findings. First, the sample size was modest, and recruitment of individuals with SCD or MCI primarily relied on participants from our previous study, many of whom were recruited online via the Dutch Brain Research Registry.^24^ This registry primarily attracts individuals who are highly educated, digitally skilled, and interested in brain health. Reflecting this, our sample largely consisted of individuals with a middle to high educational level. Additionally, most interviews were conducted via Teams, which may have further favored those comfortable with digital tools. Together, these factors may limit the generalizability of our findings to more diverse populations, including those who are less engaged, lower educated, or less digitally literate. Also, it could have possibly overestimated the prototype's positive evaluations. Many participants were already familiar with dementia risk reduction, which may partly explain their desire for more in-depth content in the prototype. Information on participants’ race or ethnicity was not collected, which may further limit insights into how cultural background influences perceptions of the prototype. In addition, most participating memory clinic professionals were women and while this reflects the current gender distribution within Dutch healthcare professions,^47^ perspectives of male professionals may be underrepresented. Second, the current study was conducted in a Dutch setting, which could limit the generalizability of our findings to other cultural or healthcare contexts. Third, the self-reported nature of SCD and MCI diagnoses may have introduced variability in the accuracy of reported diagnoses and possibly limited the representation of the broader SCD and MCI populations attending memory clinics. Fourth, all participants had high autonomy in engaging with the prototype, selecting modules and exploring the prototype at their own pace, often well in advance of the interviews. This may have led to incomplete evaluations or participants forgetting key feedback points. The short evaluation period further limits insights into the prototype's long-term effects on awareness and behavior. Future research should aim to address these limitations by incorporating larger and more diverse samples, particularly including individuals with lower digital skills or health literacy, to ensure broader applicability. Extending the evaluation period could provide insights into sustained engagement and the prototype's longer-term impact on awareness and behavior. Research should also further examine how the prototype functions in real-world clinical settings and whether it effectively enhances knowledge of dementia risk reduction, motivation, and lifestyle changes among individuals with SCD and MCI. Such evaluations are currently underway.

### Clinical implications

The BreinZorg prototype addresses previously identified needs for dementia risk reduction information and communication in memory clinic settings. For individuals with SCD or MCI, the prototype offers an accessible starting point to explore and act on brain-healthy lifestyle changes to reduce dementia risk, tailored to personal preferences. For memory clinic professionals, it provides a concrete framework to support conversations about dementia risk reduction with patients in their daily clinical practice. The prototype's dual-format design (website and hardcopy conversation aid) may improve inclusivity for individuals with limited digital skills, who are often underserved by digital-only health communication. However, this requires further validation in more diverse populations. To support future implementation, it may be beneficial to integrate the prototype into existing workflows alongside brief training or onboarding for memory clinic professionals and offer optional follow-up support for users (e.g., coaching or digital reminders). While originally developed for use in memory clinics, the prototype may also be valuable in primary care or public health settings.

### Conclusions

Individuals with SCD or MCI and memory clinic professionals considered the BreinZorg prototype to be a trustworthy and useful resource, valued its accessible design, and professionals recognized its practical application in clinical conversations. Participants appreciated its personalization features and balanced tone and expressed a desire for more in-depth content and optional follow-up support, such as coaching or reminders. Building on these results, further refinement in development and evaluation is necessary, with an emphasis on ensuring inclusivity among more diverse populations. Future research will evaluate feasibility and effects of BreinZorg among individuals with SCD or MCI in a real-world clinical setting. Furthermore, national implementation of BreinZorg is currently being explored within the Dutch Memory Clinic Network. This is with support from Alzheimer Netherlands and with continued involvement of end-users to ensure the tool remains relevant and useful.

## Supplemental Material

sj-docx-1-alz-10.1177_13872877261440966 - Supplemental material for Management of dementia risk factors by memory clinic patients and professionals: Pilot study of the BreinZorg (BrainCare) online platform Supplemental material, sj-docx-1-alz-10.1177_13872877261440966 for Management of dementia risk factors by memory clinic patients and professionals: Pilot study of the BreinZorg (BrainCare) online platform by Lotte S. Truin, Irene S. Heger, Kay Deckers, Rani Knops, Tanya Palsma, Rianne A. A. de Heus, Jurgen A. H. R. Claassen, Marjolein E. de Vugt and Sebastian Köhler in Journal of Alzheimer's Disease

sj-docx-2-alz-10.1177_13872877261440966 - Supplemental material for Management of dementia risk factors by memory clinic patients and professionals: Pilot study of the BreinZorg (BrainCare) online platform Supplemental material, sj-docx-2-alz-10.1177_13872877261440966 for Management of dementia risk factors by memory clinic patients and professionals: Pilot study of the BreinZorg (BrainCare) online platform by Lotte S. Truin, Irene S. Heger, Kay Deckers, Rani Knops, Tanya Palsma, Rianne A. A. de Heus, Jurgen A. H. R. Claassen, Marjolein E. de Vugt and Sebastian Köhler in Journal of Alzheimer's Disease

sj-docx-3-alz-10.1177_13872877261440966 - Supplemental material for Management of dementia risk factors by memory clinic patients and professionals: Pilot study of the BreinZorg (BrainCare) online platform Supplemental material, sj-docx-3-alz-10.1177_13872877261440966 for Management of dementia risk factors by memory clinic patients and professionals: Pilot study of the BreinZorg (BrainCare) online platform by Lotte S. Truin, Irene S. Heger, Kay Deckers, Rani Knops, Tanya Palsma, Rianne A. A. de Heus, Jurgen A. H. R. Claassen, Marjolein E. de Vugt and Sebastian Köhler in Journal of Alzheimer's Disease

sj-docx-4-alz-10.1177_13872877261440966 - Supplemental material for Management of dementia risk factors by memory clinic patients and professionals: Pilot study of the BreinZorg (BrainCare) online platform Supplemental material, sj-docx-4-alz-10.1177_13872877261440966 for Management of dementia risk factors by memory clinic patients and professionals: Pilot study of the BreinZorg (BrainCare) online platform by Lotte S. Truin, Irene S. Heger, Kay Deckers, Rani Knops, Tanya Palsma, Rianne A. A. de Heus, Jurgen A. H. R. Claassen, Marjolein E. de Vugt and Sebastian Köhler in Journal of Alzheimer's Disease

sj-docx-5-alz-10.1177_13872877261440966 - Supplemental material for Management of dementia risk factors by memory clinic patients and professionals: Pilot study of the BreinZorg (BrainCare) online platform Supplemental material, sj-docx-5-alz-10.1177_13872877261440966 for Management of dementia risk factors by memory clinic patients and professionals: Pilot study of the BreinZorg (BrainCare) online platform by Lotte S. Truin, Irene S. Heger, Kay Deckers, Rani Knops, Tanya Palsma, Rianne A. A. de Heus, Jurgen A. H. R. Claassen, Marjolein E. de Vugt and Sebastian Köhler in Journal of Alzheimer's Disease

sj-docx-6-alz-10.1177_13872877261440966 - Supplemental material for Management of dementia risk factors by memory clinic patients and professionals: Pilot study of the BreinZorg (BrainCare) online platform Supplemental material, sj-docx-6-alz-10.1177_13872877261440966 for Management of dementia risk factors by memory clinic patients and professionals: Pilot study of the BreinZorg (BrainCare) online platform by Lotte S. Truin, Irene S. Heger, Kay Deckers, Rani Knops, Tanya Palsma, Rianne A. A. de Heus, Jurgen A. H. R. Claassen, Marjolein E. de Vugt and Sebastian Köhler in Journal of Alzheimer's Disease
