# Supplementary material for: Management of dementia risk factors by memory clinic patients and professionals: Pilot study of the BreinZorg (BrainCare) online platform
Source: J Alzheimers Dis. 2026 Apr 15;111(3):1108–20. doi: 10.1177/13872877261440966 (PMC13219754; doi:10.1177/13872877261440966)

Supplemental Material 1 – Impression of the BreinZorg prototype

1. Images from the BreinZorg website (in Dutch)


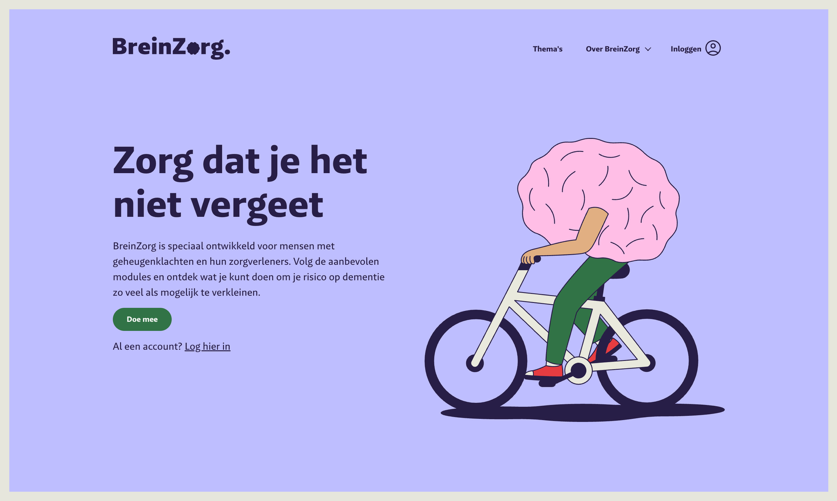


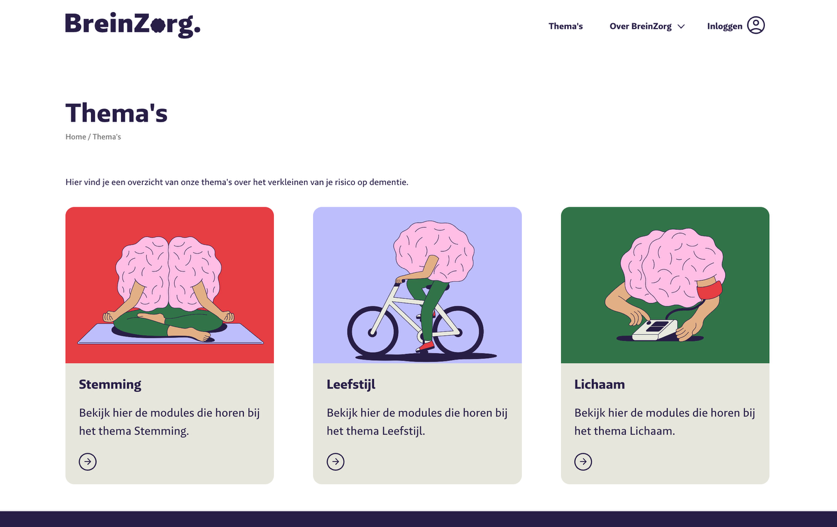


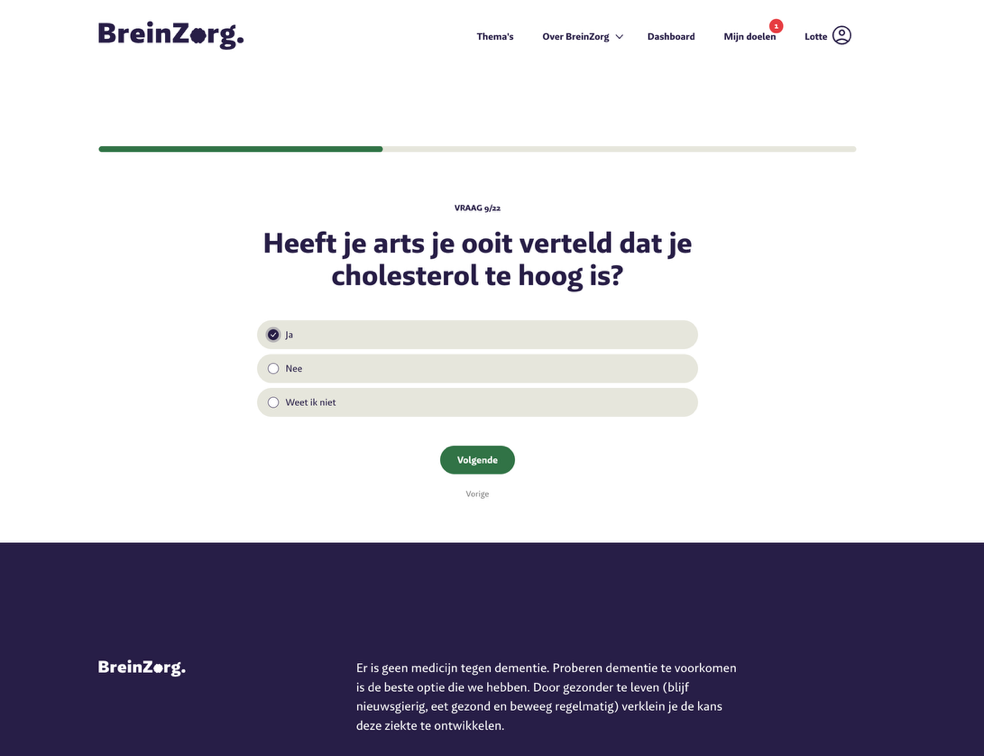

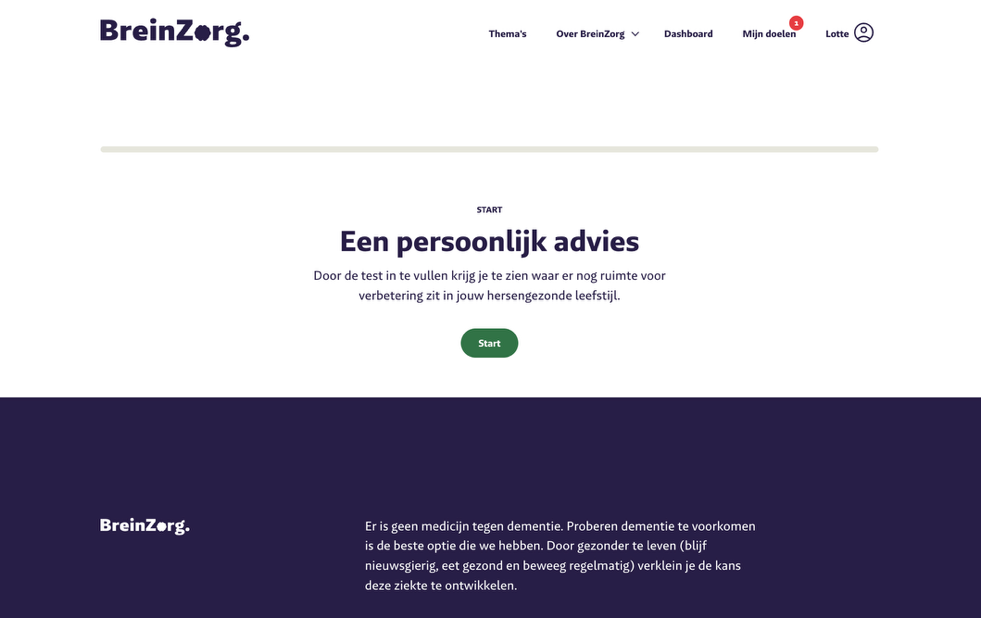


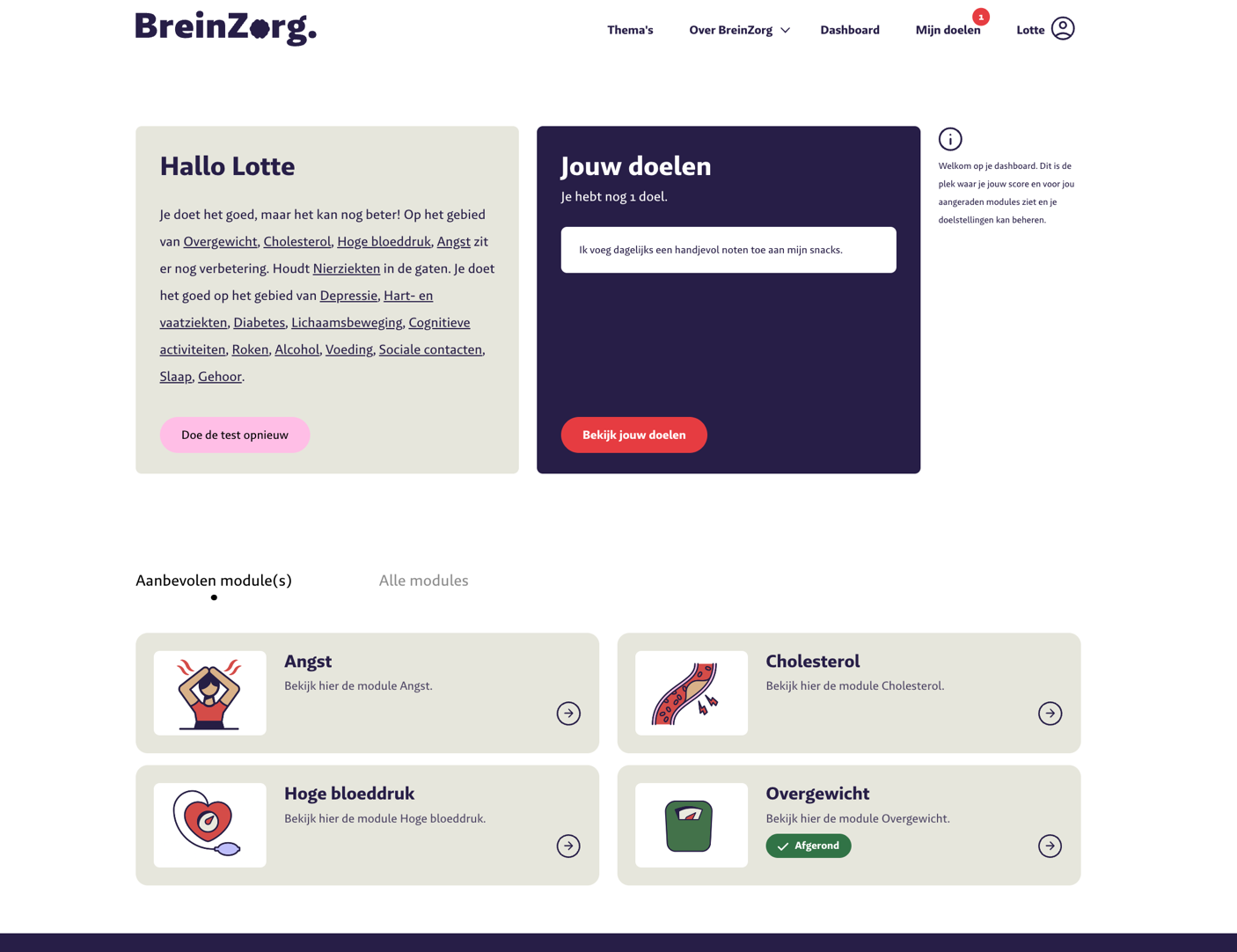


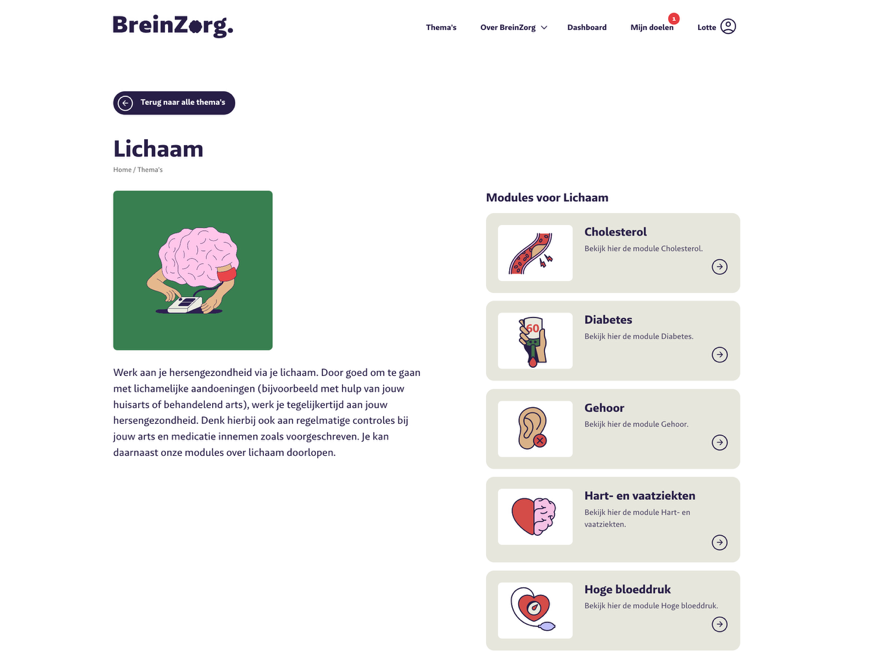


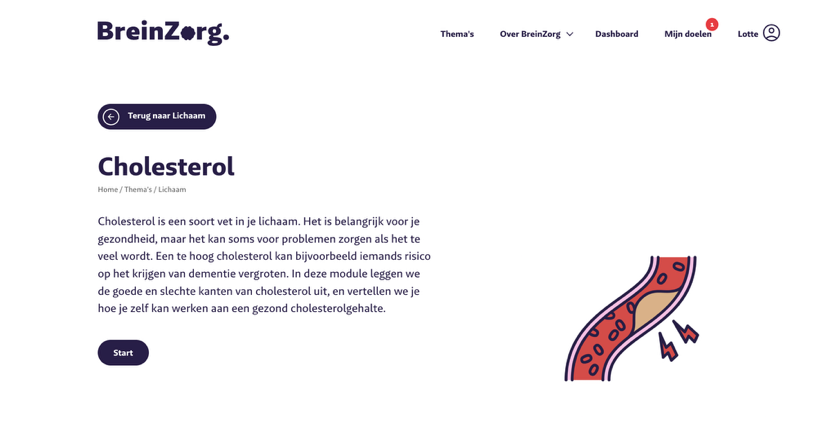

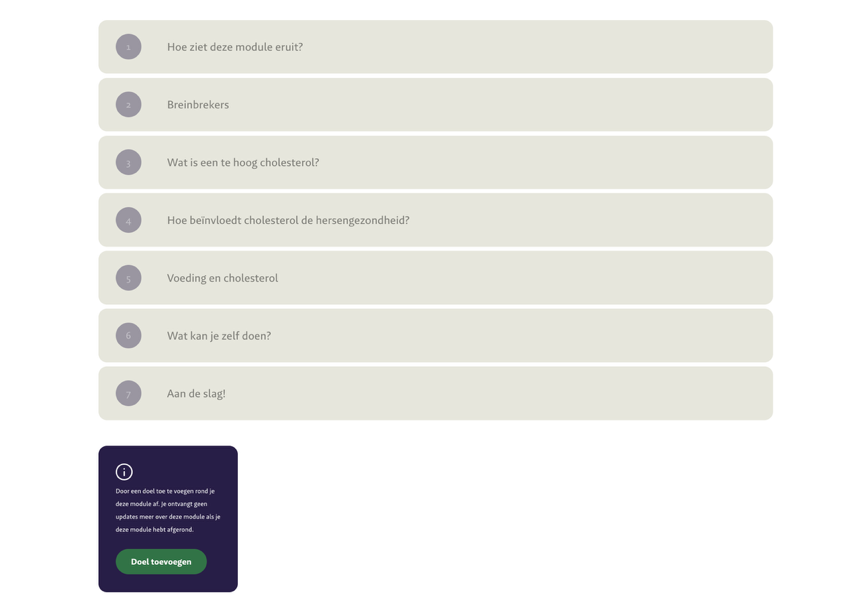


2. Images from the BreinZorg conversation aid (in Dutch)


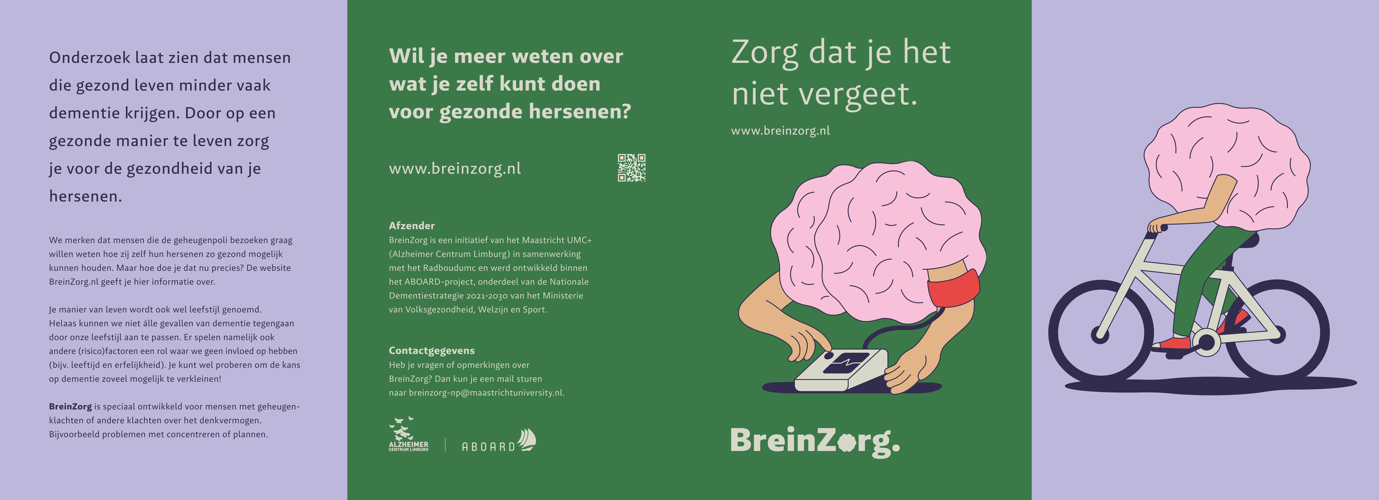


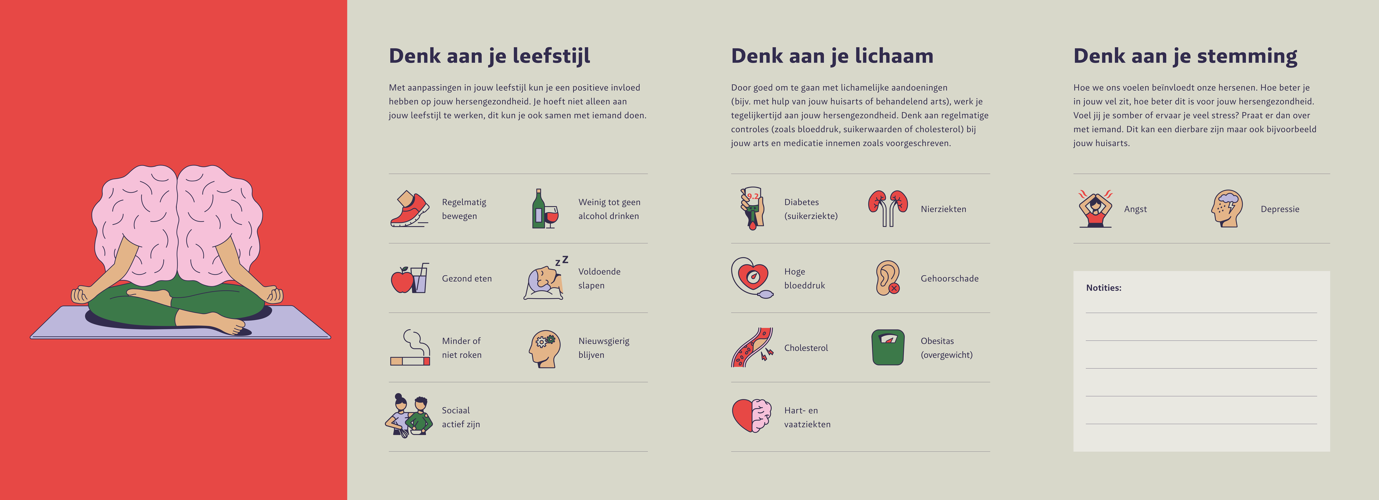


3. Images of the BreinZorg posters (in Dutch)


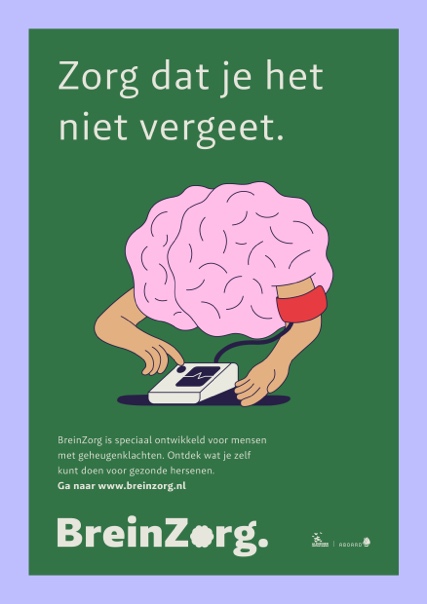

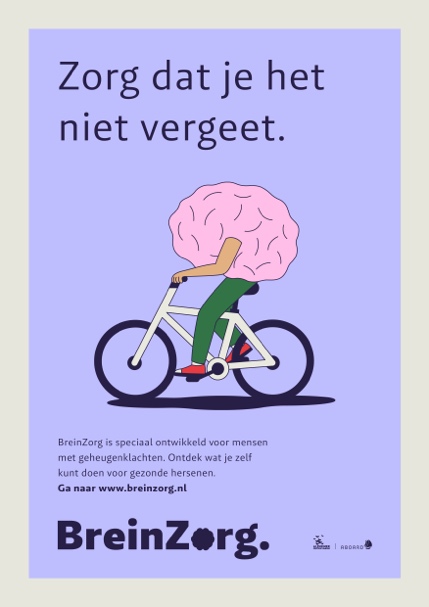

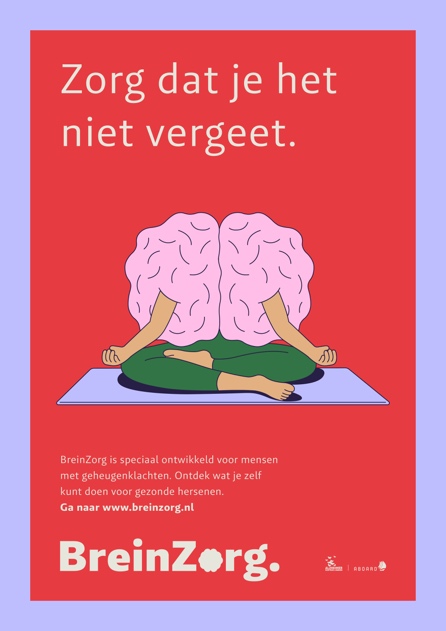

Supplement: sj-docx-1-alz-10.1177_13872877261440966 - Supplemental material for Management of dementia risk factors by memory clinic patients and professionals: Pilot study of the BreinZorg (BrainCare) online platform [file sj-docx-1-alz-10.1177_13872877261440966.docx]
