# Supplementary material for: Management of dementia risk factors by memory clinic patients and professionals: Pilot study of the BreinZorg (BrainCare) online platform
Source: J Alzheimers Dis. 2026 Apr 15;111(3):1108–20. doi: 10.1177/13872877261440966 (PMC13219754; doi:10.1177/13872877261440966)
Supplement: sj-docx-2-alz-10.1177_13872877261440966 - Supplemental material for Management of dementia risk factors by memory clinic patients and professionals: Pilot study of the BreinZorg (BrainCare) online platform [file sj-docx-2-alz-10.1177_13872877261440966.docx]

Supplemental Material 2 – Original Program Participation Questionnaire (Boots et al., 2016)

**All questions below were answered using the following scale:**

**STRONGLY DISAGREE STRONGLY AGREE**

1 | 2 | 3 | 4 | 5 | 6 | 7

| **QUESTION** | **PROMPT FOR FURTHER ELABORATION** |
| --- | --- |
| 1. I found it convenient to follow the program online | - What was (not) convenient? |
| 2. I found the website easy to use | - What was (not) easy? |
| 3. I could read the text on the website well | - Font size, contrast, amount? |
| 4. I found the instructions for using the website clear | - Why (not?)  - Suggestions for improvement? |
| 5. I found the amount of information offered to be good | - Video length, amount of text in introduction? |
| 6. I found the content of the modules clear | - Easy to understand/follow?  - Why (not)? |
| 7. I found the time I spent on a module to be good | - Approximately how much time did you spend per  module?  - What took up the most time?  - Would you have preferred to spend more/less time? |
| 8. I appreciated having conversations with a coach alongside the online program | - Why (not) appreciated?  - Added value? |
| 9. I appreciated setting goals together with the coach | - Why (not)?  - Suggestions for improvement? |
| 10. I found my coach’s verbal explanation about how to use the website clear | - Suggestions for improvement? |
| 11. I found the written explanation I received during the intake interview clear | - Suggestions for improvement? |
| 12. I felt the time between modules (1–2 weeks) was sufficient | - Shorter or longer? |
| 13. I found the number of modules (4) to be good | - More or fewer? |
| 14. I appreciated the structure of the modules (introductory video, explanation, reflection task, step-by-step plan) | - Logical structure?  - Followed all elements?  - Why (not)?  - Anything missing? |
| 15. I made use of: |  |
| 15a. the introductory videos | - Why (not)?  - What appealed/did not appeal to you? |
| 15b. the explanations | - Why (not)?  - What appealed/did not appeal to you? |
| 15c. the reflection tasks | - Why (not)?  - What appealed/did not appeal to you? |
| 15d. the step-by-step plans | - Why (not)?  - What appealed/did not appeal to you? |
| 15e. the discussion forum | - Why (not)?  - Did you post, respond to others, or read along?  - What appealed/did not appeal to you? |
| 15f. interim messages with coach via the “help”  button | - Why (not)?  - What appealed/did not appeal to you? |
|  | - Did you find it complete?  - Did you miss any functions? Suggestions?  - Would you remove certain functions? Which ones? |
| 16. I experienced privacy issues: |  |
| 16a. In general | - What did you find (un)pleasant?  - What was the reason for this? |
| 16b. During communication with my coach | - What did you find (un)pleasant?  - What was the reason for this? |
| 16c. On the discussion forum | - What did you find (un)pleasant?  - What was the reason for this? |
| 17. I am generally satisfied with what the program offered me | - Why (not)?  - Suggestions for improvement? |
| 18. The modules I took were useful for me | - Why (not)?  - Suggestions for improvement? |
| 19. I found the content of the modules interesting | - Why (not)?  - Suggestions for improvement? |
| 20. I found the tone of the text appealing | - Why (not)?  - Suggestions for improvement? |
| 21. I used the information offered in the course in my daily life | - Why (not)?  - What was the reason for this? |
| 22. After completing the course, I feel more confident that my partner and I can handle the memory problems | - Why (not)?  - What was the reason for this? |
| 23. I found the videos a good addition to the program | - Why (not)?  - Suggestions for improvement? |
| 24. I found the written explanations informative/interesting | - Why (not)?  - Suggestions for improvement? |
| 25. The reflection tasks have helped me to look more critically at my own situation | - Why (not)?  - Suggestions for improvement? |
| 26. The 5-step plan has helped me address situations I want to change in a more structured way | - Why (not)?  - Suggestions for improvement? |
| 27. I found the discussion forum a good addition to the program | - Why (not)?  - Suggestions for improvement? |
|  | - Which part(s) of the program did you find most helpful or effective, and which not? |
| 28. After completing this program, I know how to handle unwanted situations more easily in the future | - Why (not)?  - Suggestions for improvement? |
| 29. I would recommend the program to other partners of people with memory problems | - Why (not)?  - Which aspects in particular? |
| 30. Other comments? | - Is there anything else you would like to share about using the program or your satisfaction with it? |
