# Supplementary material for: Management of dementia risk factors by memory clinic patients and professionals: Pilot study of the BreinZorg (BrainCare) online platform
Source: J Alzheimers Dis. 2026 Apr 15;111(3):1108–20. doi: 10.1177/13872877261440966 (PMC13219754; doi:10.1177/13872877261440966)
Supplement: sj-docx-3-alz-10.1177_13872877261440966 - Supplemental material for Management of dementia risk factors by memory clinic patients and professionals: Pilot study of the BreinZorg (BrainCare) online platform [file sj-docx-3-alz-10.1177_13872877261440966.docx]

Supplemental Material 3 – Adapted Program Participation Questionnaire for individuals with SCD or MCI

**All questions below were answered using the following scale:**

**STRONGLY DISAGREE STRONGLY AGREE**

1 | 2 | 3 | 4 | 5 | 6 | 7

| **QUESTIONS** |
| --- |
| 1. I found it convenient to receive the information provided on the website via the internet |
| 2. I found the website easy to use |
| 3. I could read the text on the website well |
| 4. I found the tone of the text appealing |
| 5. I found the amount of information offered to be good |
| 6. I found the written explanation about how to use the website clear |
| 7. I found filling in the questionnaire burdensome |
| 8. I found filling in the questionnaire difficult |
| 9. I found the number of modules (16) to be good |
| 10. I found the content of the modules clear |
| 11. I found the content of the modules interesting |
| 12. The modules I took were useful for me |
| 13. I found the videos in the modules to be a good addition |
| 14. I appreciated the structure of the modules (introduction, in-depth information, quiz, goal setting) |
| 15. I made use of: |
| 15a. the introduction |
| 15b. the in-depth information |
| 15c. the quiz |
| 15d. the goal setting |
| 16. The quiz helped me to better understand and remember the information provided |
| 17. I appreciated setting goals independently |
| 18. I found the time I spent on a module to be good |
| 19. I experienced privacy issues |
| 20. I am generally satisfied with what was offered to me on the website |
| 21. I used the information offered on the website in my daily life |
| 22. After visiting the website, I feel more confident that I can make positive changes in my lifestyle |
| 23. After viewing the website, I know which changes I can make for a brain-healthy lifestyle |
| 24. I would recommend the website to other people with memory problems |
| 25. I found the printed materials (conversation starter, poster) to be a good supplement to the website |
| 26. I found the: |
| 26a. text on the printed materials clear |
| 26b. images on the printed materials clear |
| 26c. icons on the printed materials clear |
| 27. Other comments? |
