# Supplementary material for: Management of dementia risk factors by memory clinic patients and professionals: Pilot study of the BreinZorg (BrainCare) online platform
Source: J Alzheimers Dis. 2026 Apr 15;111(3):1108–20. doi: 10.1177/13872877261440966 (PMC13219754; doi:10.1177/13872877261440966)
Supplement: sj-docx-4-alz-10.1177_13872877261440966 - Supplemental material for Management of dementia risk factors by memory clinic patients and professionals: Pilot study of the BreinZorg (BrainCare) online platform [file sj-docx-4-alz-10.1177_13872877261440966.docx]

Supplemental Material 4 – Adapted Program Participation Questionnaire for memory clinic professionals

**All questions below were answered using the following scale:**

**STRONGLY DISAGREE STRONGLY AGREE**

1 | 2 | 3 | 4 | 5 | 6 | 7

| **QUESTIONS** |
| --- |
| 1. I found it convenient to receive the information provided on the website via the internet |
| 2. I found the website easy to use |
| 3. I could read the text on the website well |
| 4. I found the tone of the text appealing |
| 5. I found the amount of information offered to be good |
| 6. I found the written explanation about how to use the website clear |
| 7. I found filling in the questionnaire burdensome |
| 8. I found filling in the questionnaire difficult |
| 9. I found the number of modules (16) to be good |
| 10. I found the content of the modules clear |
| 11. I found the content of the modules interesting |
| 12. The modules I read were useful |
| 13. I found the videos in the modules to be a good addition |
| 14. I appreciated the structure of the modules (introduction, in-depth information, quiz, goal setting) |
| 15. I find the quiz a good addition to the modules |
| 16. I find independently setting goals a good addition to the modules |
| 17. I found the time I spent on a module to be good |
| 18. I experienced privacy issues |
| 19. I am generally satisfied with what was offered on the website |
| 20. I would use the website in my daily work to initiate conversations about lifestyle and brain health |
| 21. I believe that by visiting the website, patients will know which changes they can make for a brain-healthy lifestyle |
| 22. I would recommend the website to other people with memory problems |
| 23. I found the printed materials (conversation starter, poster) a good supplement to the website |
| 24. I found the: |
| 24a. text on the printed materials clear |
| 24b. images on the printed materials clear |
| 24c. icons on the printed materials clear |
| 25. Other comments? |
