# Supplementary material for: Management of dementia risk factors by memory clinic patients and professionals: Pilot study of the BreinZorg (BrainCare) online platform
Source: J Alzheimers Dis. 2026 Apr 15;111(3):1108–20. doi: 10.1177/13872877261440966 (PMC13219754; doi:10.1177/13872877261440966)
Supplement: sj-docx-5-alz-10.1177_13872877261440966 - Supplemental material for Management of dementia risk factors by memory clinic patients and professionals: Pilot study of the BreinZorg (BrainCare) online platform [file sj-docx-5-alz-10.1177_13872877261440966.docx]

Supplemental Material 5 – Topic list interviews on evaluation of use in clinical practice

1. How did you experience the use of BreinZorg in your daily work?
   1. Positive aspects
   2. Negative aspects
   3. Barriers to use & possible solutions
2. In what way did you use the BreinZorg materials?
   1. Was this effective?
3. At what moment did you use the BreinZorg materials?
4. Has the use of BreinZorg changed the conversation with your patient? In what way?

Which healthcare professional do you think is best suited to use this material with patients?
