# Supplementary material for: Management of dementia risk factors by memory clinic patients and professionals: Pilot study of the BreinZorg (BrainCare) online platform
Source: J Alzheimers Dis. 2026 Apr 15;111(3):1108–20. doi: 10.1177/13872877261440966 (PMC13219754; doi:10.1177/13872877261440966)
Supplement: sj-docx-6-alz-10.1177_13872877261440966 - Supplemental material for Management of dementia risk factors by memory clinic patients and professionals: Pilot study of the BreinZorg (BrainCare) online platform [file sj-docx-6-alz-10.1177_13872877261440966.docx]

Supplemental Material 6 – Adapted Technology Acceptance Model questions

**Perceived Usefulness**

1. Using BreinZorg at work would allow me to complete tasks more quickly.
2. Using BreinZorg would improve my job performance.
3. Using BreinZorg would increase my productivity.
4. Using BreinZorg would enhance my effectiveness at work.
5. Using BreinZorg would make it easier to do my job.
6. I would find BreinZorg useful in my work.

**Perceived Ease of Use**

1. Learning to use BreinZorg was easy for me.
2. I find it easy to get BreinZorg to do what I want it to do.
3. My interaction with BreinZorg is clear and smooth.
4. I find BreinZorg to be flexible to use.
5. It is easy for me to become skilled at using BreinZorg.
6. I find BreinZorg easy to use.

**7-point Likert Scale**

1 (Strongly disagree) - 2 - 3 - 4 - 5 - 6 - 7 (Strongly agree)
